# Reproductive Outcomes in Adults with 22q11.2 Deletion Syndrome

**DOI:** 10.3390/genes13112126

**Published:** 2022-11-16

**Authors:** Lisa D. Palmer, Zoë McManus, Tracy Heung, Grace McAlpine, Christina Blagojevic, Maria Corral, Anne S. Bassett

**Affiliations:** 1 The Dalglish Family 22q Clinic for Adults with 22q11.2 Deletion Syndrome, University Health Network, Toronto, ON M5G 2C4, Canada; 2 Undergraduate Medical Education, Faculty of Medicine, University of Toronto, Toronto, ON M5S 1A4, Canada; 3 Clinical Genetics Research Program, Centre for Addiction and Mental Health, Toronto, ON M5S 2S1, Canada; 4 Toronto General Hospital Research Institute, Toronto, ON M5G 2C4, Canada; 5 Campbell Family Mental Health Research Institute, Toronto, ON M5G 2C1, Canada; 6 Department of Psychiatry, University of Toronto, Toronto, ON M5S 1A4, Canada

**Keywords:** sex, stillborn, abortion, prenatal, schizophrenia, DiGeorge syndrome

## Abstract

The 22q11.2 microdeletion and its associated conditions could affect reproductive outcomes but there is limited information on this important area. We investigated reproductive outcomes in a sample of 368 adults with typical 22q11.2 deletions (median age 32.8, range 17.9–76.3 years; 195 females), and without moderate-severe intellectual disability, who were followed prospectively. We examined all reproductive outcomes and possible effects of diagnosis as a transmitting parent on these outcomes. We used logistic regression to investigate factors relevant to reproductive fitness (liveborn offspring). There were 63 (17.1%) individuals with 157 pregnancy outcomes, 94 (60.3%) of which involved live births. Amongst the remainder involving a form of loss, were seven (5.77%) stillbirths, significantly greater than population norms (*p* < 0.0001). For 35 (55.6%) individuals, diagnosis of 22q11.2 deletion syndrome (22q11.2DS) followed diagnosis of an offspring, with disproportionately fewer individuals had major congenital heart disease (CHD) in that transmitting parent subgroup. The regression model indicated that major CHD, in addition to previously identified factors, was a significant independent predictor of reduced reproductive fitness. There was evidence of persisting diagnostic delay and limited prenatal genetic testing. The findings indicate that pregnancy loss is an important health issue for adults with 22q11.2DS. CHD and/or its absence is a factor to consider in reproductive outcome research. Further studies are warranted to better appreciate factors that may contribute to reproductive outcomes, including technological advances. The results suggest the need for ongoing efforts to provide optimal education and supports to individuals with 22q11.2DS, and their clinicians, around reproductive issues and early diagnosis.

## 1. Introduction

The 22q11.2 microdeletion affects nearly one in 2000 live births and causes 22q11.2 deletion syndrome (22q11.2DS), the most common, and one of the most clinically relevant, of microdeletion syndromes [1,2]. Even though clinical testing has been available for nearly three decades [3], there continue to be diagnostic delays for many individuals, due in part to under-recognition of the condition and to the high variability in number and severity of clinical manifestations [4]. Some individuals only receive a diagnosis of this genetic condition following the diagnosis of an affected offspring that can occur any time from the prenatal period through adulthood. 

Several conditions associated with 22q11.2DS, including those present from early in development such as congenital heart disease (CHD) or intellectual disability (ID), and those that arise later such as psychotic illness, may be expected to affect reproduction [5,6]. In contrast, fertility (i.e., physical capability of reproducing) is generally considered to be unaffected in 22q11.2DS [2], and a recent study reported that the majority of adults engage in sexual activities [7]. With improved pediatric management, we can expect that an increasing number of individuals with 22q11.2DS may have children [2,8]. However, there is limited information on the full spectrum of reproductive outcomes of individuals, men and women, with the 22q11.2 microdeletion.

The aim of this study was to examine reproductive outcomes in a large, well-characterized sample of adults with 22q11.2DS where data were available on pregnancy and pregnancy outcomes. We investigated possible effects of diagnosis that occurred only after diagnosis of an offspring. We also evaluated possible predictors of live births in 22q11.2DS. Based on previous literature [5] we hypothesized that older age and absence of psychotic illness and mild ID would predict those with live births.

## 2. Materials and Methods 

### 2.1. Participants, Study Design, and Data Acquisition

The sample for this observational cohort study comprised 368 adults with 22q11.2DS (173 males, 195 females, median age 32.8, 17.9–76.3 years), all with a typical 22q11.2 deletion, confirmed by clinical genetic testing using a standard probe and fluorescence in situ hybridization (FISH) or genome-wide microarray, and followed longitudinally [9,10,11]. We excluded 38 individuals with moderate-severe ID (22 males, 16 females, median age 28.2, 19.4–63.0 years) from the initial cohort (*n* = 406), as this is a subgroup less likely to have pregnancies (and there were none in this subgroup). 

For this adult 22q11.2DS sample, we recorded lifelong data for any pregnancies among study participants or, for male participants their female partners, obtained as a routine part of initial and follow-up assessments. These included age at pregnancy, reproductive outcomes, conception method (e.g., in vitro fertilization (IVF), natural conception), and partner and relationship characteristics. Advanced maternal age was defined as ≥35 years. Stillborn was defined as the birth of a fetus at ≥20 weeks gestation, with no signs of life at birth. We also recorded whether individuals were ascertained as transmitting parents, i.e., receiving a diagnosis of 22q11.2DS through follow-up testing after determination of a 22q11.2 microdeletion in an offspring or through an affected pregnancy. As before, we examined data recorded for key phenotypic features [9,10], including level of intellectual functioning and presence of psychotic illness, and major CHD (defined as moderate to complex structural/anatomic complexity [12]), and demographic variables, sex and current age (or age at death for 40 participants, median 46.1, range 18.1–76.3 years).

The study was approved by the local research ethics boards of the Centre for Addiction and Mental Health and University Health Network, which are affiliated with the University of Toronto. Informed consent was obtained in writing for all participants.

### 2.2. Analyses

Analyses included descriptive statistics: Chi-square, Fisher’s exact, and *t*-tests, as appropriate, including those to investigate possible effects of diagnosis of 22q11.2DS as a transmitting parent. For reproductive outcomes, we searched for comparable Canadian population-based norms and identified those for stillbirths (for 1991–2020) as a proportion of all deliveries [13] to compare to those for 22q11.2DS using binomial tests. 

A logistic regression model was constructed to analyze factors of relevance to reproductive fitness in 22q11.2DS (age, male sex, mild ID, psychotic illness, and major CHD). Multicollinearity analyses to identify any potentially correlated variables in the model showed that variance inflation values did not exceed 1.5, suggesting there were no issues with multicollinearity. 

Analyses were performed using R version 4.1.3 software (R Core Team, 2022), including rms and lmtest packages for the regression analysis [14,15], or SAS 9.4 (SAS Institute, Cary, NC, USA). All statistical analyses were two-tailed with statistical significance defined as *p* values < 0.05.

## 3. Results

### 3.1. Pregnancies in Adults with 22q11.2DS

Of the 368 adults with 22q11.2DS studied, there were 63 (17.1%) with a history of at least one pregnancy. Significantly more were female (*n* = 48, 76.2% vs. *n* = 15 male, 23.8%, χ^2^ = 15.32, *p <* 0.0001). The demographic and clinical features of these 63 adults are summarized in Table 1. Their total 156 pregnancies, ranging from one to six per individual (median two, average 2.5), occurred between 1955 and 2022, with 99 in the molecular era (1994 onward). There were 154 natural and two IVF conceptions (neither of the latter involving preimplantation genetic diagnosis or a female individual with 22q11.2DS). Notably, of the 122 pregnancies occurring in 48 women with 22q11.2DS, only seven pregnancies (5.7%, in six women) involved advanced maternal age, with just one at first pregnancy. 

Examining for possible effects on pregnancy findings of diagnosis with 22q11.2DS as a transmitting parent, we found that there were 35 (55.6%) individuals in this subgroup (Table 1). None of these parental diagnoses were recorded to have followed prenatal genetic diagnostic testing or positive genetic screening for a 22q11.2 microdeletion. Those diagnosed as a transmitting parent were significantly less likely to have a major CHD (Table 1). There were no significant differences in age at first pregnancy between the diagnostic subgroups (Table 1). However, as expected given that several offspring were diagnosed as adults themselves, the transmitting parent subgroup had a significantly older current age than those receiving a diagnosis of 22q11.2DS for other reasons. Additionally, as expected, when focusing on the 44 individuals whose pregnancies were in the context of availability of molecular testing, we found that age at molecular diagnosis was significantly older for those diagnosed as transmitting parents (Table 1). The median delay between age at any pregnancy and molecular diagnosis for the transmitting parent subgroup (15 females, 6 males) was 1.9 years (range 0.4–23.5 years, with no significant between-sex difference, *p* = 0.3485). For the comparable subgroup not diagnosed as a transmitting parent (18 females, 5 males), diagnoses of 22q preceded 36 of 55 (65.5%) pregnancies. 

### 3.2. Reproductive Outcomes

There were 157 outcomes of these 156 pregnancies (one twin pregnancy resulted in one live birth and one spontaneous abortion): 94 (60.3%) live births, 34 (21.2%) spontaneous abortions, 22 (14.1%) therapeutic abortions including two late-term terminations for nonviable fetuses, and 7 (4.5%) stillbirths. 

For all nine pregnancies involving a stillbirth or otherwise fatal anomaly there were multiple features highly consistent with a 22q11.2 microdeletion. Only two of the eight from the molecular era received a molecular diagnosis of a 22q11.2 microdeletion (both postnatally). For a recent stillbirth, prenatal ultrasound findings contributed to obtaining a postnatal diagnosis and subsequent parental diagnosis. However, few of the 63 individuals in the sample with pregnancies were known to have ever had genetic prenatal testing or screening for a 22q11.2 deletion at any pregnancy [6]. As a proportion of total births over the period 1991 to 2020, stillbirths accounted for 6.4% (5 of 72) in the 22q11.2DS cohort, significantly greater than that reported for the general population (0.7%, *p* = 0.0002). In this 22q11.2DS sample, spontaneous abortions appeared consistent with the high end of Canadian population-based estimates (21.2% vs. 15–25%) [16], and therapeutic abortions were at the low end of population-based norms (23.4 vs. 28.3–32.3 per 100 live births) [17]. 

Of the 94 liveborn offspring, 28 (29.8%) were born to an individual at median age 26.6 (range 21.3–35.0) years who had been diagnosed with 22q11.2DS for reasons other than as a transmitting parent. Of these offspring, 11 (39.3%) have a 22q11.2 microdeletion. This was a significantly lower proportion than the confirmed 71.2% (47 of 66) for those born to an individual diagnosed with 22q11.2DS as a transmitting parent (χ^2^ = 9.75, *p* = 0.0018). Extending reproductive outcomes to age 2 years, there were four (4.21%) infant deaths, all with severe congenital cardiac abnormalities, two with confirmed, and two deemed retrospectively likely to have had 22q11.2 microdeletions. For the 51 parents with a 22q11.2 microdeletion who had liveborn offspring, median parental age at first live birth was 25.7 years (range 17.0–35.0) for 39 mothers, and 31.4 years (range 22.5–37.5) for 12 fathers. Twenty-seven (52.9%) of these parents with 22q11.2DS (*n* = 21, 77.8%, female) had two or more liveborn offspring; 18/27 were in the transmitting parent subgroup. 

### 3.3. Psychosocial Outcomes for Those with Liveborn Offspring 

Of the 51 adults with 22q11.DS with one or more liveborn offspring, 43 (84.3%) were married or had a long-term (>1 year) partner at the time of the birth of their first offspring; two of these were arranged marriages. Of these 43, 29 (67.4%) subsequently separated from this partner. Of the 27 individuals with two or more liveborn offspring, 17 (63.0%) had the same partner for each of these offspring. Of the 51 families where one parent had 22q11.2DS, 12 (23.5%) had received support from Child Protective Services (Children’s Aid Society in Ontario). This support ranged from one-time encounters to issues related to child custody that sometimes involved identifying an alternative living situation for the child [6,18].

### 3.4. Reproductive Fitness

To identify factors that may have an impact on reproductive fitness in 22q11.2DS, we compared the 51 (*n* = 39, 76.5% female) adults with at least one liveborn offspring to the 317 (*n* = 156, 49.2%, female) with no liveborn offspring (Table 2). There was a non-significantly lower proportion of major CHD (*p* = 0.0906) amongst those with liveborn offspring. As hypothesized, individuals with no liveborn offspring were younger, and less likely to be female, or to have a psychotic illness or mild ID (Table 2).

**Figure 1:**
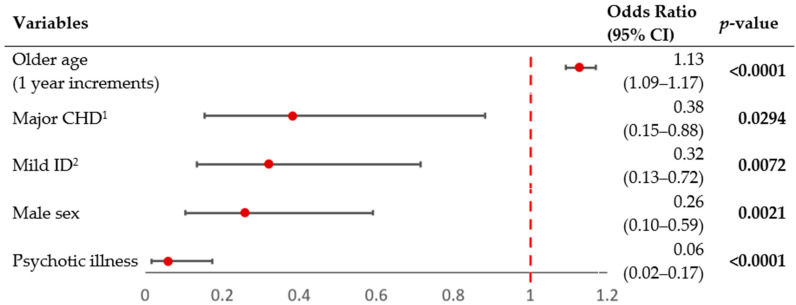
Forest plot of logistic regression model for reproductive fitness in adults with 22q11.2DS. The figure shows odds ratios (OR, red circles) and 95% confidence intervals (CI, horizontal lines) for five possible predictors of reproductive fitness in 368 adults with a 22q11.2 microdeletion. The overall logistic regression model was significant (likelihood ratio χ^2^ = 112.7, df = 5, *p <* 0.0001). Significant values are shown in bold font. ^1^ CHD = congenital heart disease. ^2^ ID = intellectual disability.

The logistic regression model to assess the likelihood of having at least one liveborn offspring was statistically significant (χ^2^= 112.7, *p <* 0.0001; Figure 1), explaining 47.7% (Nagelkerke R^2^) of the variance of this likelihood and correctly classifying 84.5% of this sample of 368 adults with 22q11.2DS. The results showed that, while accounting for effects of the other four variables, younger age, psychotic illness, male sex, mild ID, and major CHD were each independent negative predictors of having liveborn offspring in 22q11.2DS.

## 4. Discussion

The results of this study demonstrate that only a minority of women and men with 22q11.2DS have a history of pregnancy (on average 2.5 per person), even though individuals with moderate to severe intellectual disability were not included, and over half of the individuals with a pregnancy history were diagnosed to have a 22q11.2 microdeletion on the basis of a new diagnosis in an offspring. The outcomes of the 156 pregnancies involved substantial losses, including spontaneous abortions (miscarriages), stillbirths (or equivalent, fetuses with fatal anomalies), and infant deaths, most likely attributable to the 50% risk of transmitting the 22q11.2 microdeletion at each pregnancy. 

The results indicate that, in this sample, there was limited to no known uptake of available prenatal genetic testing, including that for a 22q11.2 microdeletion. There was also no indication that elective terminations were common, and indeed may be somewhat less common amongst individuals with 22q11.2DS than population norms. Direction and relative effect sizes of previous findings that male sex, major neuropsychiatric issues (psychotic illness and intellectual disability) and older age affect the likelihood of having liveborn offspring in this genetic condition were confirmed [5]. Importantly however, there were novel findings suggesting that major congenital heart disease may be an independent contributing factor to reduced reproductive fitness in 22q11.2DS. These results also suggest that studies where parents are disproportionately ascertained as transmitting parents are likely to be underpowered to detect such an effect [5]. The results for age at molecular diagnosis suggest not only historical but persisting issues with diagnostic delay and prolonged diagnostic odysseys for this under-recognized syndrome [4]. Under-detection of 22q11.2 microdeletion may be particularly likely in the absence of typical congenital cardiac anomalies [4], and thus could extend to prenatal ultrasound where these anomalies are a focus. Lack of follow-up to ensure parental testing after diagnosis in an offspring would also contribute to under-diagnosis.

For individuals with 22q11.2DS, the elevated prevalence of stillbirths, about 9-fold greater than population-based expectations, is consistent with previously reported results using an overlapping but much smaller sample (total *n* = 158) [6]. The results are also consistent with reports of elevated prevalence of 22q11.2 microdeletions in stillbirth samples assessed using genome-wide microarray [19,20]. Similarly, studies of products of conception from miscarriages indicate elevated prevalence of 22q11.2 microdeletions [20,21,22], suggesting that our results (high end of population expectations) [13] may indeed reflect some increased risk of spontaneous abortion for individuals with 22q11.2DS. 

The low uptake of prenatal genetic testing, and relatively low prevalence of elective termination, in our cohort appear comparable to previous reports [6,23]. Amongst many contributing factors, this may in part be related to the observed paucity of advanced maternal age and the under diagnosis of 22q11.2DS. Interestingly, a recent UK-based qualitative study exploring thoughts about reproductive choices of 13 adults with 22q11.2DS, including five who were parents, reflected negative views about both prenatal genetic testing and termination [24]. There is some evidence that perceptions about effects of the 22q11.2 microdeletion on a prospective offspring are likely to be shaped by the person’s own experiences of burden [24,25]. These views may contribute to some extent to the observed low overall pregnancy rate, and thus may also contribute to reproductive fitness findings.

Our findings further confirm the association of male sex and major neuropsychiatric phenotypes with lower reproductive fitness in 22q11.2DS [5], and that this is consistent with a well replicated maternal excess of inherited 22q11.2 microdeletions [5,6,26]. The findings are also consistent with lower reproductive fitness for psychotic illness and other neurodevelopmental disorders in the absence of a genetic diagnosis [27,28]. In the current study there were no individuals with moderate-severe intellectual disability by design, and mild intellectual disability appeared to have a modest but discernible effect. The novel finding that major congenital heart disease is also associated with some reduction in the likelihood of having liveborn offspring for individuals with 22q11.2DS is consistent with longstanding reports that few transmitting parents have congenital heart defects [26], as we also found in the current study, and for the general literature on major congenital heart disease reporting fewer offspring than for healthy peers [29,30,31]. 

### 4.1. Advantages and Limitations

While this study used the largest sample yet to investigate reproductive outcomes and fitness among adults with 22q11.2DS, the sample size of those with pregnancies remains relatively small. Due to diligence in tracking down elderly parents of our adults with 22q11.2DS for standard genetic testing, combined with following our cohort prospectively, the pregnancies analyzed span over six decades. The fact that over half of the individuals with pregnancies in our sample were diagnosed with 22q11.2DS following the diagnosis of an affected offspring is an ongoing ascertainment issue in the field, and applies not only to reproductive outcomes but also to other research. To our knowledge, no previous study of reproductive outcomes has reported this important ascertainment parameter. Reproductive outcomes will also be affected by temporal factors, including new technologies and their uptake [32,33], and by individual and broad cultural factors such as matchmaking [18]. There were limited population-based data available with which to compare to results for the 22q11.2DS sample. Some results for this Canadian study, where there is universal health care and access to most reproductive options, may not be generalizable to other settings. Notably, non-invasive prenatal screening (NIPS) for the 22q11.2 microdeletion [32,33] has not been an option supported by Canadian health care plans, and thus this reproductive option played no role in the current study. It is likely that prenatal ultrasound anomalies, particularly of congenital cardiac findings, may have been noted in at least some of the pregnancies of the individuals with 22q11.2DS but the current study was not designed to systematically study this. 

### 4.2. Summary, Implications and Future Directions

This study demonstrates that although individuals with a history of pregnancy are relatively uncommon, adverse reproductive outcomes, especially stillbirth, are relatively common in 22q11.2DS. Most of these adverse outcomes are likely to be related to transmission of the 22q11.2 microdeletion. Any possible role of the maternal 22q11.2DS phenotype amongst pregnant individuals, and possible effects of partner choice, genetic background, and other factors including possible interactions between maternal and fetal phenotypes, will require further study to discern. Ensuring follow-up parental testing after prenatal, pediatric and later new diagnoses of a 22q11.2 microdeletion could facilitate these studies. Confirming that male sex, and the 22q11.2DS neuropsychiatric phenotype, and also that major congenital heart disease, adversely affect reproductive fitness may prompt further study of selective advantage and disadvantage in 22q11.2DS. Such studies may benefit from comparisons with unaffected siblings. Given the numbers, it is likely that multicentre prospective studies would be necessary to better understand other factors of lower effect size that may contribute to reproductive outcomes in 22q11.2DS, such as the 22q11.2 deletion extent. 

This work supports further research to investigate factors that affect reproductive choices, pregnancy outcomes, and parenthood in 22q11.2DS. Moving forward, increased access to advanced molecular diagnostic and NIPS for 22q11.2 microdeletions [32,33], and to preimplantation genetic diagnosis, will improve our understanding of reproduction in individuals with 22q11.2DS, and will require comparison to outcomes for those with de novo 22q11.2 microdeletions. Clinically, the results emphasize the importance of supporting individuals with 22q11.2DS in making informed reproductive choices, often with supported decision-making and repeated genetic counselling, including invitations to partners and use of language that matches cognitive abilities [7,8]. Having these supports in place before, during, and after pregnancy, and with counselling and education about all aspects of reproduction, related options, and potential new roles and responsibilities as a parent, will be essential. 

In summary, results of this study have important clinical and research implications for adults with 22q11.2DS and their families, and for service providers and researchers, including those in obstetrics and maternal fetal medicine. Further research is needed about reproductive outcomes for individuals with 22q11.2 microdeletion and other rare genomic disorders that are increasingly detectable.

## Figures and Tables

**Table 1:** Demographic and clinical variables for 63 adults with 22q11.2DS with a history of pregnancy assessing possible effects of diagnosis as a transmitting parent.

Demographic/Clinical Variables	Adults with 22q11.2DS and History of Pregnancy (*n* = 63)	Adults with 22q11.2DS and Diagnosis as a Transmitting Parent		Chi-Square/Fisher’s/Wilcoxon Two-Sample Test	
		Yes (*n* = 35)	No (*n* = 28)		
	*n* (%)			χ^2^	*p*-Value
Male sex	15 (23.8)	10 (28.6)	5 (17.9)	0.98	0.3211
Major congenital heart disease	15 (23.8)	1 (2.9)	14 (50.0)	19.06	**<0.0001**
Psychotic illness	11 (17.5)	4 (11.4)	7 (25.0)	-	0.1935
Mild intellectual disability	18 (28.6)	8 (22.9)	10 (35.7)	1.26	0.2617
	**Median (Range)**			Z	
Current age	42.4 (24.7–76.3)	50.0 (28.9–69.5)	37.9 (24.7–76.3)	3.54	**0.0002**
Age at first pregnancy	26.2 (17.0–38.0)	26.0 (17.0–38.0)	26.3 (19.7–31.0)	1.20	0.1157
Age at molecular diagnosis ^1^	23.6 (0.1–52.5)	32.3 (20.7–52.5)	16.3 (0.1–39.6)	4.83	**<0.0001**

^1^ Age at molecular diagnosis is provided only for the 44 individuals with pregnancies that occurred in the molecular era (*n* = 21 in the diagnosis as a transmitting parent subgroup, and *n* = 23 in the not diagnosed as a transmitting parent subgroup), involving a total of 96 pregnancies. For the remaining 19 individuals, the median age at molecular diagnosis was 43.0 (range 25.6–72.4) years. Bold font indicates statistically significant findings.

**Table 2:** Adults with 22q11.2DS (*n* = 368) comparing those with (*n* = 51) and without (*n* = 317) liveborn offspring.

Demographic/Clinical Variables	Live Offspring (*n* = 51)	No Live Offspring (*n* = 317) ^1^	Chi-Square/Wilcoxon Two-Sample Test	
	*n* (%)	*n* (%)	χ^2^	*p*-Value
Mild intellectual disability	13 (25.5)	181 (57.1)	16.36	**<0.0001**
Male sex	12 (23.5)	161 (50.8)	12.03	**0.0005**
Psychotic illness	7 (13.7)	116 (36.6)	9.32	**0.0023**
Major congenital heart disease	11 (21.6)	110 (34.7)	2.86	0.0906
	**Median (Range)**		Z	
Current age	45.8 (24.7–76.3)	30.5 (17.9–67.6)	6.94	**<0.0001**

^1^ Includes *n* = 12 individuals with a history of pregnancy. Bold font indicates statistically significant findings.

## Data Availability

The data are not publicly available due to ethical restrictions and privacy concerns. Any data requests can be directed to the corresponding author. StatsCan stillbirth information is available at: https://www150.statcan.gc.ca/t1/tbl1/en/tv.action?pid=1310042801, Doi: https://doi.org/10.25318/1310042801-eng, accessed on 10 October 2022.
